# Supplementary material for: A preclinical model of chronic pancreatitis driven by trypsinogen autoactivation
Source: Nat Commun. 2018 Nov 28;9:5033. doi: 10.1038/s41467-018-07347-y (PMC6261995; doi:10.1038/s41467-018-07347-y)

SUPPLEMENTARY INFORMATION

A preclinical model of chronic pancreatitis driven by trypsinogen autoactivation

Andrea Geisz and Miklós Sahin-Tóth

Supplementary Figure 1. The recombined *T7D23A* targeting sequence. The 5' homology arm starts at c.-4286 in the 5' upstream region and the 3' homology arm ends at c.595-136 in intron 4. Exons are highlighted in yellow, mutation c.68A>C (p.D23A) in exon 2 is in red, the neomycin cassette in intron 1 (between nucleotides c.41-346 and c.41-345) is in blue and the loxP sites are in magenta. Relative to the reference sequence, we found a c.-1110C>A variation, indicated in gray, lowercase bold type. Splice sites are underlined and italicized.

GTGCTCATCTGCGTTTCCTGTGGCCTCAGGGCACAACAAAGCCAGAAAAGGCCATTGGTGAAGGTACAGCCTCACTG
GCAGTTGAAGGCCCAGGACTGAAAGGGTCATGAAGAGAAGTTTAGGCTTAGCACCATGAAAAGAGCACATGATAGGC
TATTTGTCAAAGTGCAGCCCAGTTGCAGCAGAAGACAGCAATGTTTTGGAGATGTCAGTACCATGAGATGACCACCA
AGAACAATAATAGCAGCAGTACAGTACAGGCTGCTGGAGCCTAGAAGACACAAGATGTTTGCTCTAAAGAGCAGAGC
TGGAGAAGTGACCCAAGCCCTTGGAGGAGCCCAAAAGATCATGAGTGGATCTCAAACATTGGATTGTTCAAGTTTGA
TTTTGCTTGTTCTGTGCCCTGATATTTTTCCCTGTTGAAGCAAGAAAGTATTTTAATGGAGCTCACAGTTAAGAGAC
TTTGAATTGTAAAAATACATTGAATTTCAAAAGATACTGGATATTTTAAAGGGATTGAAATTTTAATATGTAAGAAT
TTGTAAAGATTGTGGGACTTTTAAAACTATTTAGATCTTGGGGATGAATAAGAAAGTAAGAGTTGAAGCTTAATAGT
GATGAGTTTGTGTGTCAAGTTGACAATGGATCAGTTGTACTGGTTGGTTTTGTGTGTCAACTTGACACAAGCTGGAG
TTATCACAGAAAAAGGAACCTCCTTTGAGGAAATGCCTCCATGAAATCCAGCTGTGAGGCACTTTTTTCAATTAGTA
ATCAAGGGTGGGAGGGCCCATTGTGGGTGGTGCCATCCCTGGGCTTGTAGTCCTAGGTTCAATAAGAAAGCAAGCTG
AGCAAGCCAGGGAAAGCAAGCCAGTAAGGAATATCTCTCCATGGCCTCTGCATCAGCTCCTGCTTCCTGACCTGCTT
AAGTTCCAGTTCTGACTTCCTTTGGTGATGAAAAGCAATGTGGAAGTGTAAGCTGAATAAATCCTTTCCTCCTCAAC
ATGCTTCTTGGTCAGGATGTTTGTGCAGGACTAGAAGCCCTGACTAAGACATGTTGGAAGAGTTTTGCTTGGAGAGG
ACACTTGAAAACTTGGCATTGGTTGTCACAAATGCAAGAAAGAGGTAGTGCTCTTTCTATATCAAGATTCTGGTAAA
CACAGGAGTTTCCTATCACAAAGACTTAAGAGGCTCCAAATGTCAATAGTGTCAAAGTTGAGAATCTCTTTTCCACA
TATTATATATAATATATTACTTCGTCACAGCGACCAAAGTATCAAACACAGATACATTAAGAAATGAAAGTGTAATG
GCTCATGGCTTAAAATGTTCTTTTCATCATGTGAAGAAATGCATAACAGAGAGGAGCCATTATTGCAAGCCAGGAAG
CTGAAGAGGAGAAGCCAGGCACTGCACTGACTCTCTCCTTTCTTCACGTCTGTCATCTTGGGCCTCCAACCCAGCAA
TGGCACCAACTACATTCAAGTAGGGTTTTCCCTATCAGTTAACTGTCTCTGGAAATGACCTCATACACAGTGCCAGT
ATTGTAACCCTTCAAATTCCTAGATAATTCTAAACCCCATCATGTTGATAATGAAGATTCGCCATCCCAACTAAGTT
GTCTTGGTAACAGCAAAATTTTAGAACAAGTCTTCAAGAATGACATTTTAAACAAAAATAACATACTGGTATTTGAG
TTGAGATTTTTAGACAGGTAGAGAATCTGACTATTGTCTTTAGCTAGAGAGTTACTTTGTATGAAGAATCCCTCTAA
CCAGACATGGTAGTGCACATCTCTAATCCCATTCATCCTGGGACTGTGAGTTTGAATCTAACCTGGGAAATTTTAAA
ACTGTCTCAAAATAAACATTTATAAAGGATGGAAGTGACAGGAAAAATGGTACATGCCTTTTATCCCAGCACCCAGA
AAAATAAGGTAGGCAGACACCAATGACTTTAAGGGCAGCTAGTCTATATAAAGACCAGCTCTAGGCCAGTTCCAGGG
GTACATAGTAAGACTCTGTCTTAAGCAAAAGTGGAAAGGGTCTTGTCGATTAGATTAGCTTAGCTTAGTGATAGAAC
ATGAACTTTCTGGTCTAGCATAGTGAGGCCCTGGGTTCAACTCCAAATACTGCATAGGAGAAAAGCTTGCCTAACTC
TTGATACTTGACTCTTCCATTAATTCTTATAATTTCTGCTTCAAAAATGCAATTATTGAAAAATCTATGAACTCAAT
AATATTCACTTAACTTGGTAGTTCATTCTGTGTGCCTTATGTTTAATATATAATGCTGCATAATTCTGCTAGGAGAC
TGCCTTGCAATTTACACATCTGGTTCATGAAGTTTTCATCTGAAACCTGGGAATTAAAATAGGTATATCAAATCAAT
CTTCTTAGGAGGGAGCACTAAATTAATATAAGCTTTTTCTATGTTTATGCATGTTTATATACATATATATGTTTATA
TACAGATATATGTTTTCCTATATACATGTTTATATACATATAAATGTTTTCCTATAAATCCTTATGATAAAATTTAG
TTTACAAATAAAAAAGTTAAGAGATTGAAACAATGAGTCATAAAATGTAATAGCAATAAAATGGAAGAAATCAATGA
TATGCAATGACATAGTTATATTAACATTATGAATTTCAGTAATTTTAAAAATAATTTTGGTTCATGTATCCATGGAC
TGAAGTCAATTGGAAACACGGGCAAAGGAAGCACGGAAAAGGGAACCGCCATATCCTAGATTAGAACAAGCTCTAGA
TAAACACACACAACTTCTCACCTTTAACTTTACCATTTTTTAATTATTTTTTAATTCTTTTCTACAGTCCAGACTTC
ACCCCTCCCAGTCTTCCCTACAACCACTCCCCATCCCATACCTCCTCCCCCCTATCTCCAAGAGTATGTTCCCAACT
CCACCCCTACTCCACCAGGCCTCCCCACTCCCTGGGGCCCAAGTCTCTGGGGTTTAGGTGCATCTTCTCTCACTGAG
GTCAGTCCAGAGAGTCCTTTGCTGTATATGTATACAGGGTCTCATATCAGCTGGTGTATGCTGCCTGGTTGATGGCT
CAGTGTCTGAGAGATCTCAGGGTTCCAGGTCAGTTGAGACTGCTGGTCTTCCCATGGAGCTGCCCTCTCCTCAGCTT
CTTCCAGCTGTTTCCCAAG**a**CAACCACAGGGGTCTCAGGCTTCTGTTCACTGGTTGGGTGCTAGTATCTGCATCTGA
CTCTTTCAGCTGCTTGTTGGGCTTCTCAGAGGGCAGTCATGCTAGGCTCCTGTCTGTAAGCACACCATAGCATCAGT
AACAGTTTCAGGGTCCCAGGCCTCCCTTTGAACTTGATCCTAATTTGGGCCTTTCACTGGACCTCCTTTCTCTCATG
CTCATCTCCATTTTTTGTCCCTGCAGATCTTTCTGACAGGAACAATTCTGGGTCAGAGTTTTTGACTATGGGATGGC

AACCCCATCCCTCATCTGATGTCCTGTCTTTCTACTGGATGTGGACTCTGCAAGTTCCCTCTCCCCACTGTTGGGAT
TCTCATCTAAGGTCCCTCTCTTTGAGTTCTAAGAATCTCTCACCTCCCAGGTCTCCTGTACATTCTAGAGGGACCCC
TACATCCTACCTCCCGAGGTTGCCTGTTTCCATTCTTCCTGATGGCCCTCAGGGCTTCAGTTCTATTACCCCCAATA
CCTGATCATATTTCCCCCTTCCCTTCCCTGTCCTCTCTCCTACCCAGGTCCCTCCCACCTTCTGTCCCCAGTGACTG
CTTTCATTAAAGAAGAGTTTTCCTTTCCTTGAAAGAGAAGGTCTTTTACATTAGCAATGGTGTCTTAATGGGGATGT
AGAGTACTAGAAGGGCCCTAGGAAAAGTTATACATTTTTAATAATTACAAAATAGACATTGTCATGTGAAAGACCAG
AGTTCCAATGAGTCCTTTATAAAAATGCTTACATCTGGAATGTTAGAGTACCTAAGACTTTTGTCTGAGAACCTGAA
AACCTGAAGTTTTGCCTTGTGACTTTACAAACTTTTTTTCCTACTATGGAGAACAGTTCCCACCAGGTGAGTTTGTT
TCTTCCATTCCCACACCCTTTAGATTTTTGTCCTGCATTATCTTCCATCTTTCACTGATTTGAATTCTAAATTTAGA
TTTCTGAGTTTCCAATGAAAGAACTTTTCTGGCACCCATGGCCATGGGTGTCAACAAGACATGAAGGTATAAATAGC
TGTGGAGGAAAGCCTTCATCCTATTGACTGCTCTAGCCACAGTGAGCAACC**ATGAAGACCTTAATCTTCCTTGCCTT**
CCTTGGAGCTGCTG_GTGAGT_ACCTTTAATGAATAAGTCACTAAGAGTTAACCATCTTTGCTCTTTGGTTGGTGGTTC
AATCTCTGGAAACCCCCAAGGATCCAAGTACAAGGAAGGTGTTTTGGACTAAACTCAAGACTTAGTGAATACCAGGA
AAATATTCACCACTTCCAACATATATAATCCCTATTTTTTATATAAATTTATTTATTAAATGCATGTGAGTACACTG
TCCAATCTTCTGACACACCAGAAGAGGGCATCAGATCCCATTACAGATGGTTGTGAGCCACCATGTGGTTGCTGGGA
GTTGAACTCAGGACCTCTGGAAGACCAGTCAGTGCTCTTTCCACTGAGCCATCTCTCCAGCCCATATGATGCCTGTT
TTACTTTTAATTTTCATAATGAGTCTTGCTAAATCGTCCAAGCTACCACTCAGCACATTCTATCGCCCAGACAGATC
TTAAGTCTGCCTCATCCACCAAAGCAGTGGAGTGGGAACTACAGGTCTGAGACAAAATGCAAGGATGCAATTAAAAT
TGCAGCACTGAAAGGTAATATGAGTTTCTCCAGAACTCAGGAAGTATAAACTAGACATTTCTCTCAATACTTCCAGA
AATATCTTAGCTAATAAAATGTCTTAGCTCAGAAAAATAATGTAAGACCTTGAAACTAACAGTGGACCCTACTACAC
AATGCAGGTTCGAAGTCGACCTGCAGCCAAGCTATCGAATTCCTGCAGCCCAATTCCGATCATATTCAATAACCCTT
AATATAACTTCGTATAATGTATGCTATACGAAGTTATTAGGTCTGAAGAGGAGTTTACGTCCAGCCAAGCTAGCTTG
GCTGCAGGTCGTCGAAATTCTACCGGGTAGGGGAGGCGCTTTTCCCAAGGCAGTCTGGAGCATGCGCTTTAGCAGCC
CCGCTGGGCACTTGGCGCTACACAAGTGGCCTCTGGCCTCGCACACATTCCACATCCACCGGTAGGCGCCAACCGGC
TCCGTTCTTTGGTGGCCCCTTCGCGCCACCTTCTACTCCTCCCCTAGTCAGGAAGTTCCCCCCCGCCCCGCAGCTCG
CGTCGTGCAGGACGTGACAAATGGAAGTAGCACGTCTCACTAGTCTCGTGCAGATGGACAGCACCGCTGAGCAATGG
AAGCGGGTAGGCCTTTGGGGCAGCGGCCAATAGCAGCTTTGCTCCTTCGCTTTCTGGGCTCAGAGGCTGGGAAGGGG
TGGGTCCGGGGGCGGGCTCAGGGGCGGGCTCAGGGGCGGGGCGGGCGCCCGAAGGTCCTCCGGAGGCCCGGCATTCT
GCACGCTTCAAAAGCGCACGTCTGCCGCGCTGTTCTCCTCTTCCTCATCTCCGGGCCTTTCGACCTGCAGCCTGTTG
ACAATTAATCATCGGCATAGTATATCGGCATAGTATAATACGACAAGGTGAGGAACTAAACC**ATG**GGATCGGCCATT
GAACAAGATGGATTGCACGCAGGTTCTCCGGCCGCTTGGGTGGAGAGGCTATTCGGCTATGACTGGGCACAACAGAC
AATCGGCTGCTCTGATGCCGCCGTGTTCCGGCTGTCAGCGCAGGGGCGCCCGGTTCTTTTTGTCAAGACCGACCTGT
CCGGTGCCCTGAATGAACTGCAGGACGAGGCAGCGCGGCTATCGTGGCTGGCCACGACGGGCGTTCCTTGCGCAGCT
GTGCTCGACGTTGTCACTGAAGCGGGAAGGGACTGGCTGCTATTGGGCGAAGTGCCGGGGCAGGATCTCCTGTCATC
TCACCTTGCTCCTGCCGAGAAAGTATCCATCATGGCTGATGCAATGCGGCGGCTGCATACGCTTGATCCGGCTACCT
GCCCATTCGACCACCAAGCGAAACATCGCATCGAGCGAGCACGTACTCGGATGGAAGCCGGTCTTGTCGATCAGGAT
GATCTGGACGAAGAGCATCAGGGGCTCGCGCCAGCCGAACTGTTCGCCAGGCTCAAGGCGCGCATGCCCGACGGCGA
TGATCTCGTCGTGACCCATGGCGATGCCTGCTTGCCGAATATCATGGTGGAAAATGGCCGCTTTTCTGGATTCATCG
ACTGTGGCCGGCTGGGTGTGGCGGACCGCTATCAGGACATAGCGTTGGCTACCCGTGATATTGCTGAAGAGCTTGGC
GGCGAATGGGCTGACCGCTTCCTCGTGCTTTACGGTATCGCCGCTCCCGATTCGCAGCGCATCGCCTTCTATCGCCT
TCTTGACGAGTTCTTC**TGA**GGGGATCAATTCTCTAGAGCTCGCTGATCAGCCTCGACTGTGCCTTCTAGTTGCCAGC
CATCTGTTGTTTGCCCCTCCCCCGTGCCTTCCTTGACCCTGGAAGGTGCCACTCCCACTGTCCTTTCCTAATAAAAT
GAGGAAATTGCATCGCATTGTCTGAGTAGGTGTCATTCTATTCTGGGGGGTGGGGTGGGGCAGGACAGCAAGGGGGA
GGATTGGGAAGACAATAGCAGGCATGCTGGGGATGCGGTGGGCTCTATGGCTTCTGAGGCGGAAAGAACCAGCTGGG
GCTCGACTAGAGCTTGCGGAACCCTTAATATAACTTCGTATAATGTATGCTATACGAAGTTATTAGGTCCCTCGAGA
TTTAAATAGTCCATGTTGCAAAGGTTGTTGGCTTAAATGAAAAGGTTAGTAAGGGAAATCTTGTCATGCTTGGTGAA
ATTGTTTATAAAAACTTCAGTTACAAGCAATAAGAGAGATAATCAGAATAAAAACATTAAACATTTTTTCTTTAAAG
TGAGAATACTTTCAGCCTGGTCCTTAAGTTTTTCTTCTTTGCTTATGGAGATAAACATGTCATGCCAACAACTTATC
GCCTGCCAAGATTAACGCAAAGCTATCCATTTGCATGGCTTCTCAGCAGATTGTGTTATAAACTGTCCCTTATGTCC
TCAAATAGTCACAATCAGGCATCTCTGGCTGACTTTCTTCA_CAG_**TTGCTCTCCCCCTGGATGATGATGATGCCAAAA**
TTGTTGGAGGCTACACCTGCCAGAGGAATGCTCTCCCCTACCAGGTGTCTCTGAACAGCGGCTACCATTTTTGTGGA
GGCTCACTCATCAATTCCCAGTGGGTTGTTTCAGCCGCTCACTGCTACAAATC_GTAAGT_GACCAATATTCACATTTC
CATGCTCTAAATCCATTGTCCTTTGGTCTTATCAAAATACAATTAGGAAATGTGCACAGTTATATAGGATATTTAAA
ACGTCCAGATACTGGATTGTAGAGATGGATCAGTTGTTGAGAACACTTGCTGCTCTTCTGGAAGGCCTGGGTTTGAT
TCTAAGCAAAAAAATGGTTCACAACTACCTGTAACCCTAGTGTCATGGGATCTTATACCTTCTTCTGGATTCCACTG
GGCATCTGCATTCATACATGAACATACCTACACACAGACATGTATGCATGTATATAATTAAAAGTAAATTTAAAAAA
AATCAGATATCAAGAACTTGATTCTATCTCAACAATTATATGATCTACTGAGGGACATTTTTATGGAGTGTTGTTCC

AGGATTCATTACTATCTTTCTAGGTGATGATAGTAAAGACCCAGAAGGGAACCATCATTTTTTAAGGATGTCTTAGA
ATAGATTCAGATTGTGAATCATCATATAGCTTTAACAAAGGGAAATGGGCATGTAAAGATAAAACACTATATTTTAA
ATACATTATGAAAAACAAGCATACATAACTCTGTCCTATAACATTGCTCTGCTTTTCAGCCAAGTGTGGTGGTACAC
ACCTTTAATCCCAGCACTTCTTTTCTGACATTTTGTTTCATCCCACTGTTTATTTATCTTAATATCTTAAATCCTTG
TATATCTTATATATCTAAAGTCTTGAAGAATTACAATTGGAAAAATAGCAAACACCAACCAATCTGTTCTTCATCTC
CAACTACTTAGTAAGAATGCTTCTTCCCATCTTATATCTTGTATTTTAATAGATAATGAACTGTGAGTCTGTACTAA
CGAGGAGGGAAATAATTCCAAGGAATTTTCTGACCTACAAGCAGGAAGGGAGTGCACACTTACCTAGGATATAAAAT
CACCTATGTGAATATTTGTGTTTATTTGCATGATTGGAAAATTGGGGGAAGGGAAAGATTGAAGTTGGATATGTTAA
AAACTGCTAATCCTTATATC*CAG***CCGAATTCAGGTGCGCCTGGGAGAACACAACATTGATGCCCTTGAGGGTGGTGA**
GCAATTCATTGATGCAGCTAAAATCATCCGCCACCCCAACTATAATGCAAACACCTATAACAATGACATCATGTTGA
TTAAGCTGAAGACAGCTGCCACCCTCAACTCTCGAGTGTCCACTGTCGCTCTGCCAAGATCTTGTCCATCAGCTGGT
ACTAGGTGTCTTGTGTCTGGCTGGGGCAACACCCTGAGCTCTGGCA*GTAAGT*ACCACTGGGACTTGATTACCAAGTG
TGAGAAGACTAGAGATGAAAAGCACGGTGGGGGGCAAGGTGGTCCAAATTCTCACTCAAGGGAAGTTTCTCTAAGGA
GAGAGGCATATGTAGGCAGTGTTAACATGCATAAATTAGATAGAAAACTTCTGCCCACATCATACAATAGAGACAAC
AAAAATGAACTTGTAGAAAAAAACACTCAAAAGCATTTAACTGAAATGGGGGAAGTAAGTAAACAGTGGCTAAAGAT
TTTGGTGTTCCTTTAAGTATACACTTGTTGAAAGCGGTGGACACCAATGCTCGCACCTGATTTTTAAAAAAAAAAAA
TGTTGTGAATTGAATAAAGTTACTTTCTCAAAAAATGTTCTCCGAAATTATAAGTCACTGGGGAATTTAAAGACAGA
GTAGGTGTTAAGAAAGAGGCAGAAGGCTGGGAAAGTTGTTTCCAAAAACCATGGATAATCTATTTTCATTCTTGTGT
TTGA*CAG***CCAACTACCCTTCACTCCTTCAGTGTCTTGATGCTCCTGTCCTCTCTGACAGTTCTTGCACAAGTTCTTA**
CCCAGGCAAGATCACTAGCAACATGTTCTGTCTGGGCTTCCTGGAGGGCGGAAAGGACTCCTGCCAG*GTGGGT*TGAT
TTGTGGTTGTGCTCCCTCTTATTCATTCTCCCTCCTCCCTCTGCATCTTTGAAAAAAAAAAAAGATGGAGAGAGAACA
TTAACCATCTCTTAGACTATTGGTAATAGTAGATTTGGAATATAAATTTAATTGTCTCTATTATATTGATGACTAGA
ACTAAATGTAAAAAAGATATACAGAAATTACTACAAACATTTTATCTGACCGTGTACCTCAAAAGCTTTATTATAGA
CTTTACCTTTGAAAGAAGAGCAGAACATTTGAAAAAGTATTCAGTCTCCCCAGTTCTCACACATTTGTAGGTTGATA
GATTGACTAAAGAAAGGTGTAGGTCTGTAAGAAAGATAAAGAACAGTGGCATACAAGGAGACTGCAG

Supplementary Figure 2. The recombined *T7D23A* sequence after Cre-mediated deletion of the neomycin cassette. The 5' homology arm starts at c.-4286 in the 5' upstream region and the 3' homology arm ends at c.595-136 in intron 4. Exons are highlighted in yellow, mutation c.68A>C (p.D23A) in exon 2 is in red, the residual scar sequence in intron 1 (between nucleotides c.41-346 and c.41-345) is in blue and the remaining loxP site is in magenta. Relative to the reference sequence, we found a c.-1110C>A variation, indicated in gray, lowercase bold type. Splice sites are underlined and italicized.

The primers used for genotyping are also indicated.

Forward primer: CTT GAA ACT AAC AGT GGA CCC T

Reverse primer: AAC TGT GCA CAT TTC CTA ATT G

Products: Mutant allele 757 bp, wild-type allele 630 bp

```
GTGCTCATCTGCGTTTCCTGTGGCCTCAGGGCACAACAAAGCCAGAAAAGGCCATTGGTGAAGGTACAGCCTCACTG
GCAGTTGAAGGCCCAGGACTGAAAGGGTCATGAAGAGAAGTTTAGGCTTAGCACCATGAAAAGAGCACATGATAGGC
TATTTGTCAAAGTGCAGCCCAGTTGCAGCAGAAGACAGCAATGTTTTGGAGATGTCAGTACCATGAGATGACCACCA
AGAACAATAATAGCAGCAGTACAGTACAGGCTGCTGGAGCCTAGAAGACACAAGATGTTTGCTCTAAAGAGCAGAGC
TGGAGAAGTGACCCAAGCCCTTGGAGGAGCCCAAAAGATCATGAGTGGATCTCAAACATTGGATTGTTCAAGTTTGA
TTTTGCTTGTTCTGTGCCCTGATATTTTTCCCTGTTGAAGCAAGAAAGTATTTTAATGGAGCTCACAGTTAAGAGAC
TTTGAATTGTAAAAATACATTGAATTTCAAAAGATACTGGATATTTTAAAGGGATTGAAATTTTAATATGTAAGAAT
TTGTAAAGATTGTGGGACTTTTAAAACTATTTAGATCTTGGGGATGAATAAGAAAGTAAGAGTTGAAGCTTAATAGT
GATGAGTTTGTGTGTCAAGTTGACAATGGATCAGTTGTACTGGTTGGTTTTGTGTGTCAACTTGACACAAGCTGGAG
TTATCACAGAAAAAGGAACCTCCTTTGAGGAAATGCCTCCATGAAATCCAGCTGTGAGGCACTTTTTTCAATTAGTA
ATCAAGGGTGGGAGGGCCCATTGTGGGTGGTGCCATCCCTGGGCTTGTAGTCCTAGGTTCAATAAGAAAGCAAGCTG
AGCAAGCCAGGGAAAGCAAGCCAGTAAGGAATATCTCTCCATGGCCTCTGCATCAGCTCCTGCTTCCTGACCTGCTT
AAGTTCCAGTTCTGACTTCCTTTGGTGATGAAAAGCAATGTGGAAGTGTAAGCTGAATAAATCCTTTCCTCCTCAAC
ATGCTTCTTGGTCAGGATGTTTGTGCAGGACTAGAAGCCCTGACTAAGACATGTTGGAAGAGTTTTGCTTGGAGAGG
ACACTTGAAAACTTGGCATTGGTTGTCACAAATGCAAGAAAGAGGTAGTGCTCTTTCTATATCAAGATTCTGGTAAA
CACAGGAGTTTCCTATCACAAAGACTTAAGAGGCTCCAAATGTCAATAGTGTCAAAGTTGAGAATCTCTTTTCCACA
TATTATATATAATATATTACTTCGTCACAGCGACCAAAGTATCAAACACAGATACATTAAGAAATGAAAGTGTAATG
GCTCATGGCTTAAAATGTTCTTTTCATCATGTGAAGAAATGCATAACAGAGAGGAGCCATTATTGCAAGCCAGGAAG
CTGAAGAGGAGAAGCCAGGCACTGCACTGACTCTCTCCTTTCTTCACGTCTGTCATCTTGGGCCTCCAACCCAGCAA
TGGCACCAACTACATTCAAGTAGGGTTTTCCCTATCAGTTAACTGTCTCTGGAAATGACCTCATACACAGTGCCAGT
ATTGTAACCCTTCAAATTCCTAGATAATTCTAAACCCCATCATGTTGATAATGAAGATTCGCCATCCCAACTAAGTT
GTCTTGGTAACAGCAAAATTTTAGAACAAGTCTTCAAGAATGACATTTTAAACAAAAATAACATACTGGTATTTGAG
TTGAGATTTTTAGACAGGTAGAGAATCTGACTATTGTCTTTAGCTAGAGAGTTACTTTGTATGAAGAATCCCTCTAA
CCAGACATGGTAGTGCACATCTCTAATCCCATTCATCCTGGGACTGTGAGTTTGAATCTAACCTGGGAAATTTTAAA
ACTGTCTCAAAATAAACATTTATAAAGGATGGAAGTGACAGGAAAAATGGTACATGCCTTTTATCCCAGCACCCAGA
AAAATAAGGTAGGCAGACACCAATGACTTTAAGGGCAGCTAGTCTATATAAAGACCAGCTCTAGGCCAGTTCCAGGG
GTACATAGTAAGACTCTGTCTTAAGCAAAAGTGGAAAGGGTCTTGTCGATTAGATTAGCTTAGCTTAGTGATAGAAC
ATGAACTTTCTGGTCTAGCATAGTGAGGCCCTGGGTTCAACTCCAAATACTGCATAGGAGAAAAGCTTGCCTAACTC
TTGATACTTGACTCTTCCATTAATTCTTATAATTTCTGCTTCAAAAATGCAATTATTGAAAAATCTATGAACTCAAT
AATATTCACTTAACTTGGTAGTTCATTCTGTGTGCCTTATGTTTAATATATAATGCTGCATAATTCTGCTAGGAGAC
TGCCTTGCAATTTACACATCTGGTTCATGAAGTTTTCATCTGAAACCTGGGAATTAAAATAGGTATATCAAATCAAT
CTTCTTAGGAGGGAGCACTAAATTAATATAAGCTTTTTCTATGTTTATGCATGTTTATATACATATATATGTTTATA
TACAGATATATGTTTTCCTATATACATGTTTATATACATATAAATGTTTTCCTATAAATCCTTATGATAAAATTTAG
TTTACAAATAAAAAAGTTAAGAGATTGAAACAATGAGTCATAAAATGTAATAGCAATAAAATGGAAGAAATCAATGA
TATGCAATGACATAGTTATATTAACATTATGAATTTCAGTAATTTTAAAAATAATTTTGGTTCATGTATCCATGGAC
TGAAGTCAATTGGAAACACGGGCAAAGGAAGCACGGAAAAGGGAACCGCCATATCCTAGATTAGAACAAGCTCTAGA
```

TAAACACACACAACTTCTCACCTTTAACTTTACCATTTTTTAATTATTTTTTAATTCTTTTCTACAGTCCAGACTTC
ACCCCTCCCAGTCTTCCCTACAACCACTCCCCATCCCATACCTCCTCCCCCCTATCTCCAAGAGTATGTTCCCAACT
CCACCCCTACTCCACCAGGCCTCCCCACTCCCTGGGGCCCAAGTCTCTGGGGTTTAGGTGCATCTTCTCTCACTGAG
GTCAGTCCAGAGAGTCCTTTGCTGTATATGTATACAGGGTCTCATATCAGCTGGTGTATGCTGCCTGGTTGATGGCT
CAGTGTCTGAGAGATCTCAGGGTTCCAGGTCAGTTGAGACTGCTGGTCTTCCCATGGAGCTGCCCTCTCCTCAGCTT
CTTCCAGCTGTTTCCCAAG**a**CAACCACAGGGGTCTCAGGCTTCTGTTCACTGGTTGGGTGCTAGTATCTGCATCTGA
CTCTTTCAGCTGCTTGTTGGGCTTCTCAGAGGGCAGTCATGCTAGGCTCCTGTCTGTAAGCACACCATAGCATCAGT
AACAGTTTCAGGGTCCCAGGCCTCCCTTTGAACTTGATCCTAATTTGGGCCTTTCACTGGACCTCCTTTCTCTCATG
CTCATCTCCATTTTTTGTCCCTGCAGATCTTTCTGACAGGAACAATTCTGGGTCAGAGTTTTTGACTATGGGATGGC
AACCCCATCCCTCATCTGATGTCCTGTCTTTCTACTGGATGTGGACTCTGCAAGTTCCCTCTCCCCACTGTTGGGAT
TCTCATCTAAGGTCCCTCTCTTTGAGTTCTAAGAATCTCTCACCTCCCAGGTCTCCTGTACATTCTAGAGGGACCCC
TACATCCTACCTCCCGAGGTTGCCTGTTTCCATTCTTCCTGATGGCCCTCAGGGCTTCAGTTCTATTACCCCCAATA
CCTGATCATATTTCCCCCTTCCCTTCCCTGTCCTCTCTCCTACCCAGGTCCCTCCCACCTTCTGTCCCCAGTGACTG
CTTTCATTAAAGAAGAGTTTTCCTTTCCTTGAAAGAGAAGGTCTTTTACATTAGCAATGGTGTCTTAATGGGGATGT
AGAGTACTAGAAGGGCCCTAGGAAAAGTTATACATTTTTAATAATTACAAAATAGACATTGTCATGTGAAAGACCAG
AGTTCCAATGAGTCCTTTATAAAAATGCTTACATCTGGAATGTTAGAGTACCTAAGACTTTTGTCTGAGAACCTGAA
AACCTGAAGTTTTGCCTTGTGACTTTACAAACTTTTTTTCCTACTATGGAGAACAGTTCCCACCAGGTGAGTTTGTT
TCTTCCATTCCCACACCCTTTAGATTTTTGTCCTGCATTATCTTCCATCTTTCACTGATTTGAATTCTAAATTTAGA
TTTCTGAGTTTCCAATGAAAGAACTTTTCTGGCACCCATGGCCATGGGTGTCAACAAGACATGAAGGTATAAATAGC
TGTGGAGGAAAGCCTTCATCCTATTGACTGCTCTAGCCACAGTGAGCAACC**ATGAAGACCTTAATCTTCCTTGCCTT**
CCTTGGAGCTGCTG*GTGAGT*ACCTTTAATGAATAAGTCACTAAGAGTTAACCATCTTTGCTCTTTGGTTGGTGGTTC
AATCTCTGGAAACCCCCAAGGATCCAAGTACAAGGAAGGTGTTTTGGACTAAACTCAAGACTTAGTGAATACCAGGA
AAATATTCACCACTTCCAACATATATAATCCCTATTTTTTATATAAATTTATTTATTAAATGCATGTGAGTACACTG
TCCAATCTTCTGACACACCAGAAGAGGGCATCAGATCCCATTACAGATGGTTGTGAGCCACCATGTGGTTGCTGGGA
GTTGAACTCAGGACCTCTGGAAGACCAGTCAGTGCTCTTTCCACTGAGCCATCTCTCCAGCCCATATGATGCCTGTT
TTACTTTTAATTTTCATAATGAGTCTTGCTAAATCGTCCAAGCTACCACTCAGCACATTCTATCGCCCAGACAGATC
TTAAGTCTGCCTCATCCACCAAAGCAGTGGAGTGGGAACTACAGGTCTGAGACAAAATGCAAGGATGCAATTAAAAT
TGCAGCACTGAAAGGTAATATGAGTTTCTCCAGAACTCAGGAAGTATAAACTAGACATTTCTCTCAATACTTCCAGA
AATATCTTAGCTAATAAAATGTCTTAGCTCAGAAAAATAATGTAAGAC**CTTGAAACTAACAGTGGACCCT**ACTACAC
AATGCAGGTTCGAAGTCGACCTGCAGCCAAGCTATCGAATTCCTGCAGCCCAATTCCGATCATATTCAATAACCCTT
AATATAACTTCGTATAATGTATGCTATACGAAGTTATTAGGTCCCTCGAGATTTAAATAGTCCATGTTGCAAAGGTT
GTTGGCTTAAATGAAAAGGTTAGTAAGGGAAATCTTGTCATGCTTGGTGAAATTGTTTATAAAAACTTCAGTTACAA
GCAATAAGAGAGATAATCAGAATAAAAACATTAAACATTTTTTCTTTAAAGTGAGAATACTTTCAGCCTGGTCCTTA
AGTTTTTCTTCTTTGCTTATGGAGATAAACATGTCATGCCAACAACTTATCGCCTGCCAAGATTAACGCAAAGCTAT
CCATTTGCATGGCTTCTCAGCAGATTGTGTTATAAACTGTCCCTTATGTCCTCAAATAGTCACAATCAGGCATCTCT
GGCTGACTTTCTTCA*CAG***TTGCTCTCCCCCTGGATGATGATGATGCCAAAATTGTTGGAGGCTACACCTGCCAGAGG**
AATGCTCTCCCCTACCAGGTGTCTCTGAACAGCGGCTACCATTTTTGTGGAGGCTCACTCATCAATTCCCAGTGGGT
TGTTTCAGCCGCTCACTGCTACAAATC*GTAAGT*GACCAATATTCACATTTCCATGCTCTAAATCCATTGTCCTTTGG
TCTTATCAAAATA**CAATTAGGAAATGTGCACAGTT**ATATAGGATATTTAAAACGTCCAGATACTGGATTGTAGAGAT
GGATCAGTTGTTGAGAACACTTGCTGCTCTTCTGGAAGGCCTGGGTTTGATTCTAAGCAAAAAAATGGTTCACAACT
ACCTGTAACCCTAGTGTCATGGGATCTTATACCTTCTTCTGGATTCCACTGGGCATCTGCATTCATACATGAACATA
CCTACACACAGACATGTATGCATGTATATAATTAAAAGTAAATTTAAAAAAAATCAGATATCAAGAACTTGATTCTA
TCTCAACAATTATATGATCTACTGAGGGACATTTTTATGGAGTGTTGTTCCAGGATTCATTACTATCTTTCTAGGTG
ATGATAGTAAAGACCCAGAAGGGAACCATCATTTTTTAAGGATGTCTTAGAATAGATTCAGATTGTGAATCATCATA
TAGCTTTAACAAAGGGAAATGGGCATGTAAAGATAAAACACTATATTTTAAATACATTATGAAAAACAAGCATACAT
AACTCTGTCCTATAACATTGCTCTGCTTTTCAGCCAAGTGTGGTGGTACACACCTTTAATCCCAGCACTTCTTTTCT
GACATTTTGTTTCATCCCACTGTTTATTTATCTTAATATCTTAAATCCTTGTATATCTTATATATCTAAAGTCTTGA
AGAATTACAATTGGAAAAATAGCAAACACCAACCAATCTGTTCTTCATCTCCAACTACTTAGTAAGAATGCTTCTTC
CCATCTTATATCTTGTATTTTAATAGATAATGAACTGTGAGTCTGTACTAACGAGGAGGGAAATAATTCCAAGGAAT
TTTCTGACCTACAAGCAGGAAGGGAGTGCACACTTACCTAGGATATAAAATCACCTATGTGAATATTTGTGTTTATT
TGCATGATTGGAAAATTGGGGGAAGGGAAAGATTGAAGTTGGATATGTTAAAAACTGCTAATCCTTATATC*CAG***CCG**
AATTCAGGTGCGCCTGGGAGAACACAACATTGATGCCCTTGAGGGTGGTGAGCAATTCATTGATGCAGCTAAAATCA
TCCGCCACCCCAACTATAATGCAAACACCTATAACAATGACATCATGTTGATTAAGCTGAAGACAGCTGCCACCCTC
AACTCTCGAGTGTCCACTGTCGCTCTGCCAAGATCTTGTCCATCAGCTGGTACTAGGTGTCTTGTGTCTGGCTGGGG
CAACACCCTGAGCTCTGGCA*GTAAGT*ACCACTGGGACTTGATTACCAAGTGTGAGAAGACTAGAGATGAAAAGCACG
GTGGGGGGCAAGGTGGTCCAAATTCTCACTCAAGGGAAGTTTCTCTAAGGAGAGAGGCATATGTAGGCAGTGTTAAC
ATGCATAAATTAGATAGAAAACTTCTGCCCACATCATACAATAGAGACAACAAAAATGAACTTGTAGAAAAAAACAC

TCAAAAGCATTTAACTGAAATGGGGGAAGTAAGTAAACAGTGGCTAAAGATTTTGGTGTTCCTTTAAGTATACACTT
GTTGAAAGCGGTGGACACCAATGCTCGCACCTGATTTTTAAAAAAAAAAAATGTTGTGAATTGAATAAAGTTACTTT
CTCAAAAAATGTTCTCCGAAATTATAAGTCACTGGGGAATTTAAAGACAGAGTAGGTGTTAAGAAAGAGGCAGAAGG
CTGGGAAAGTTGTTTCCAAAAACCATGGATAATCTATTTTCATTCTTGTGTTTGA*CAG***CCAACTACCCTTCACTCCT**
TCAGTGTCTTGATGCTCCTGTCCTCTCTGACAGTTCTTGCACAAGTTCTTACCCAGGCAAGATCACTAGCAACATGT
TCTGTCTGGGCTTCCTGGAGGGCGGAAAGGACTCCTGCCAG*GTGGGT*TGATTTGTGGTTGTGCTCCCTCTTATTCAT
TCTCCCTCCTCCCTCTGCATCTTTGAAAAAAAAAAAGATGGAGAGAGAACATTAACCATCTCTTAGACTATTGGTAA
TAGTAGATTTGGAATATAAATTTAATTGTCTCTATTATATTGATGACTAGAACTAAATGTAAAAAAGATATACAGAA
ATTACTACAAACATTTTATCTGACCGTGTACCTCAAAAGCTTTATTATAGACTTTACCTTTGAAAGAAGAGCAGAAC
ATTTGAAAAAGTATTCAGTCTCCCCAGTTCTCACACATTTGTAGGTTGATAGATTGACTAAAGAAAGGTGTAGGTCT
GTAAGAAAGATAAAGAACAGTGGCATACAAGGAGACTGCAG

Supplementary Figure 3. Uncropped version of western blots shown in Figure 1F.

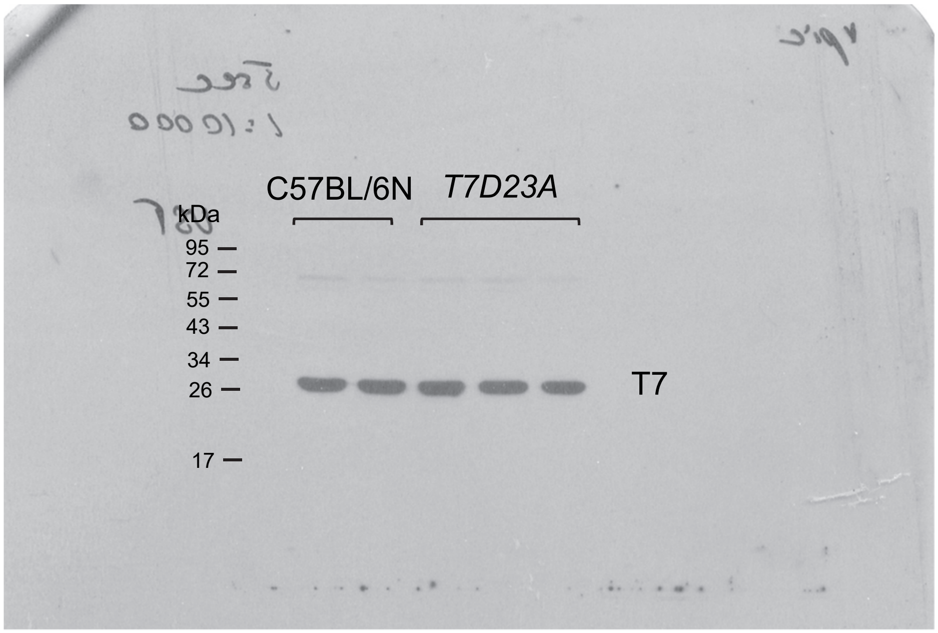

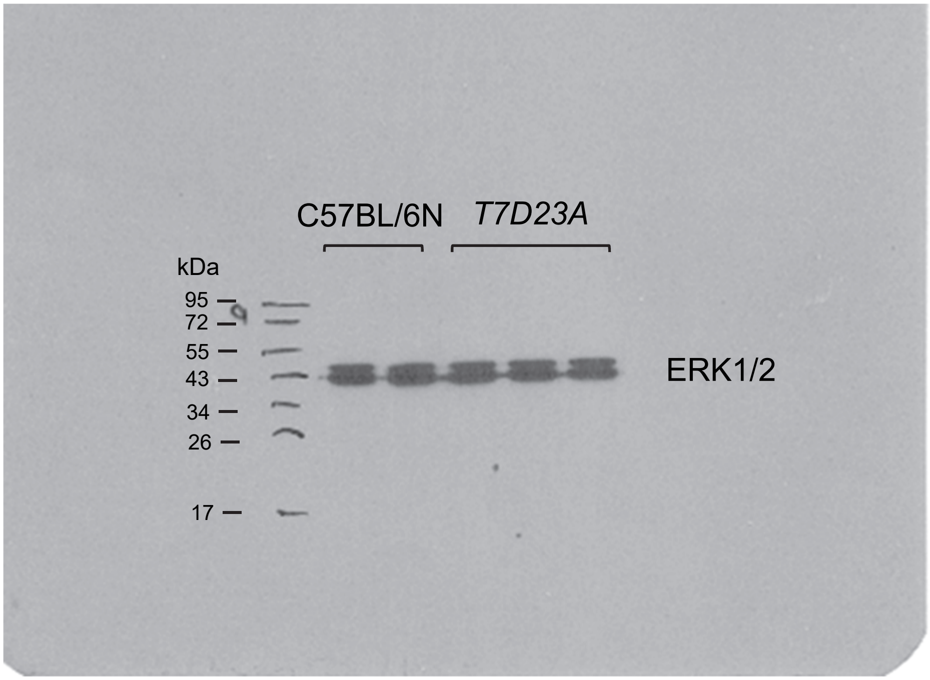

Supplement: Supplementary file 1 — Supplementary Information [file 41467_2018_7347_MOESM1_ESM.pdf]
